# Supplementary material for: The Role of the Dorsolateral Prefrontal Cortex in Ego Dissolution and Emotional Arousal During the Psychedelic State
Source: Hum Brain Mapp. 2025 Apr 9;46(5):e70209. doi: 10.1002/hbm.70209 (PMC11979361; doi:10.1002/hbm.70209)
Supplement: Supplementary file 1 — Figure S1. Delta analysis reveals regions of the brain that become less functionally connected to the left DLPFC on LSD. Blue areas denote regions that decrease in RSFC on LSD; they include the left precentral gyrus, right supramarginal gyrus, left inferior frontal gyrus, and left fusiform gyrus. Figure S2. Results of the exploratory tests on all three seeds with the 11‐dimensional ASC questionnaire results. (a) RSFC between the rDLPFC seed and the left putamen is positively associated with changed meaning of percepts. (b) RSFC between the lDLPFC seed and the left lingual gyrus is negatively associated with anxiety. Figure S3. A scatterplot of the correlation between ego dissolution and the RSFC in the initial and confirmational analyses. The x‐axis is the changes in RSFC between placebo and LSD. The y‐axis is the demeaned VAS measures of Ego Dissolution taken post scan. Figure S4. A scatterplot of the correlation between Emotional Arousal and the RSFC in the initial and confirmational analyses. The x‐axis is the changes in RSFC between placebo and LSD. The y‐axis is the demeaned VAS measures of Emotional Arousal taken post scan. [file HBM-46-e70209-s001.docx]

**Supplementary Figures**


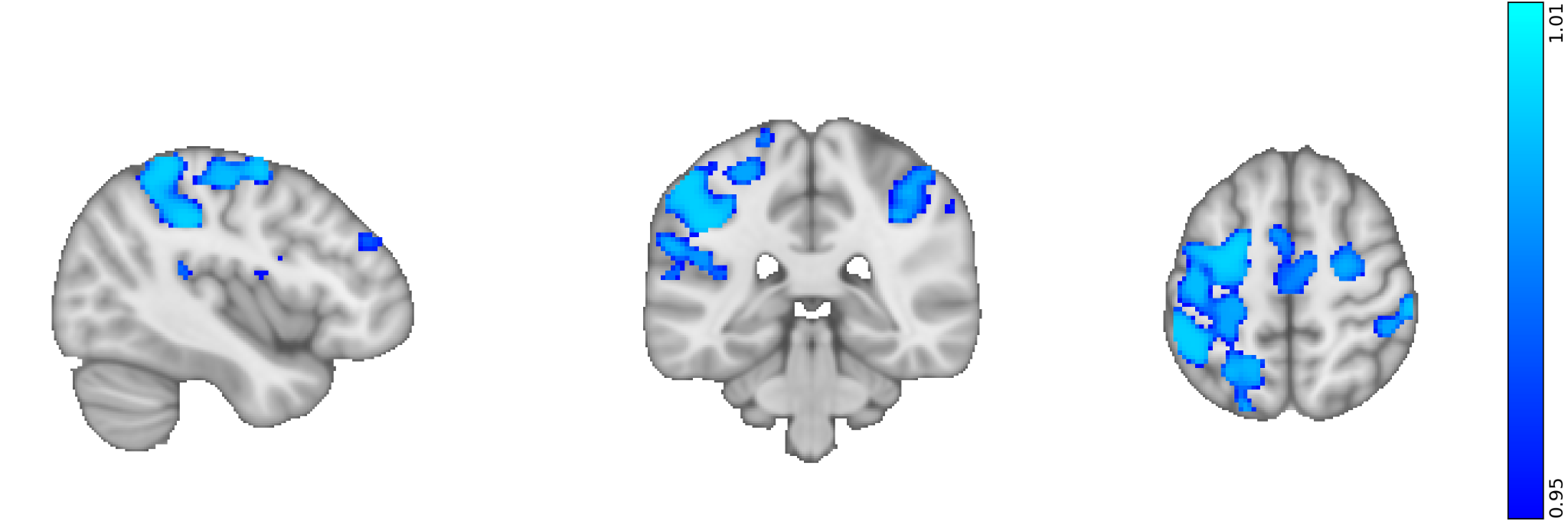


**Figure S1: Delta analysis reveals regions of the brain that become less functionally connected to the left DLPFC on LSD. Blue areas denote regions that decrease in RSFC on LSD; they include the left precentral gyrus, right supramarginal gyrus, left inferior frontal gyrus, and left fusiform gyrus.**

**
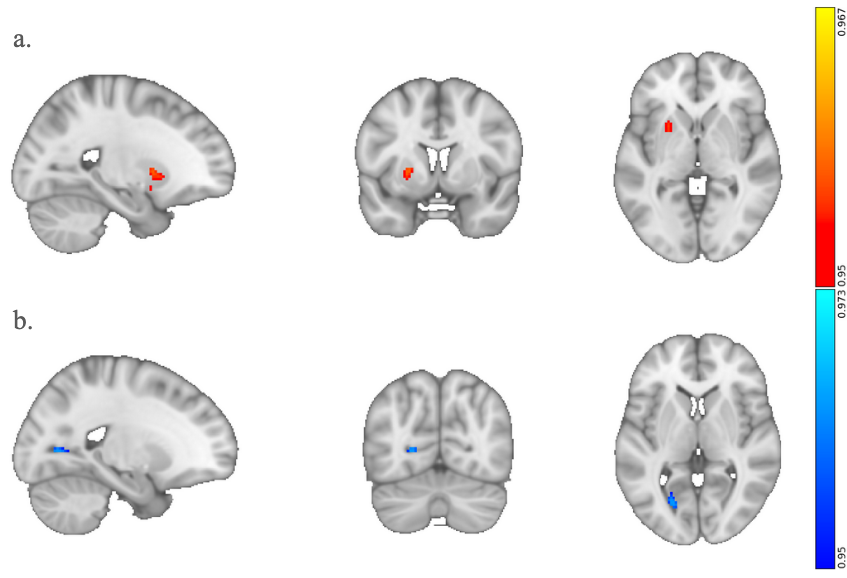
**

**Figure S2: Results of the exploratory tests on all three seeds with the 11-dimensional ASC questionnaire results. a) RSFC between the rDLPFC seed and the left putamen is positively associated with changed meaning of percepts. b) RSFC between the lDLPFC seed and the left lingual gyrus is negatively associated with anxiety.**


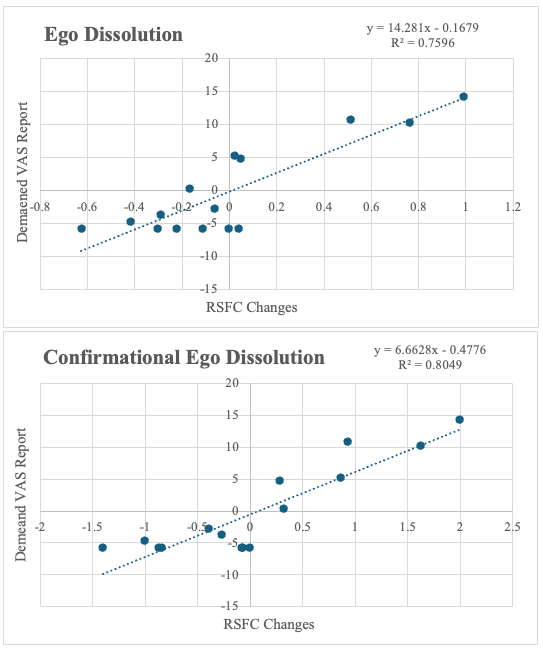


**Figure S3: A scatterplot of the correlation between ego dissolution and the RSFC in the initial and confirmational analyses. The x-axis is the changes in RSFC between placebo and LSD. The y-axis is the demeaned VAS measures of Ego Dissolution taken post scan.**


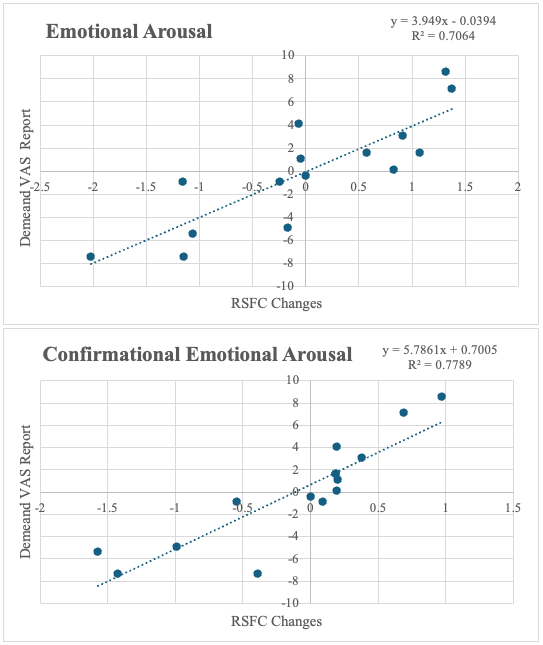


**Figure S4: A scatterplot of the correlation between Emotional Arousal and the RSFC in the initial and confirmational analyses. The x-axis is the changes in RSFC between placebo and LSD. The y-axis is the demeaned VAS measures of Emotional Arousal taken post scan.**
